# Supplementary material for: Angelica sinensis polysaccharide as potential protectants against recurrent spontaneous abortion: focus on autophagy regulation
Source: Front Med (Lausanne). 2025 Jan 15;12:1522503. doi: 10.3389/fmed.2025.1522503 (PMC11774876; doi:10.3389/fmed.2025.1522503)
Supplement: Supplementary material S1 — The main instruments used during the LC-MS process, along with their models/specifications and manufacturers. [file Supplementary_file_1.zip › Metabolomics sequencing data FC1.2/8.代谢通路分析/Exposure_Ctrl/all/KEGG/mmu02010.html]

KEGG PATHWAY: ABC transporters - Mus musculus (house mouse)


# ABC transporters - Mus musculus (house mouse)


[
Pathway menu
| Organism menu
| Pathway entry
| Show description
| Download
| Help
]

The ATP-binding cassette (ABC) transporters form one of the largest known protein families, and are widespread in bacteria, archaea, and eukaryotes. They couple ATP hydrolysis to active transport of a wide variety of substrates such as ions, sugars, lipids, sterols, peptides, proteins, and drugs. The structure of a prokaryotic ABC transporter usually consists of three components; typically two integral membrane proteins each having six transmembrane segments, two peripheral proteins that bind and hydrolyze ATP, and a periplasmic (or lipoprotein) substrate-binding protein. Many of the genes for the three components form operons as in fact observed in many bacterial and archaeal genomes. On the other hand, in a typical eukaryotic ABC transporter, the membrane spanning protein and the ATP-binding protein are fused, forming a multi-domain protein with the membrane-spanning domain (MSD) and the nucleotide-binding domain (NBD).


##### Option

Scale:


100%

Image resolution:


 High

##### Search

##### ID search

##### Color

##### Related Brite

- 02000  Transporters


KGML

Image (png) file 1x
